# GLIPR1 modulates the response of cisplatin-resistant human lung cancer cells to cisplatin

**DOI:** 10.1371/journal.pone.0182410

**Published:** 2017-08-03

**Authors:** Xin Gong, Jing Liu, Dan Zhang, Dawei Yang, Zhihui Min, Xiaoxing Wen, Guifang Wang, Huayin Li, Yuanlin Song, Chunxue Bai, Jing Li, Jian Zhou

**Affiliations:** 1 Department of Pulmonary Medicine, Shanghai Respiratory Research Institute, Zhongshan Hospital, Fudan University, Shanghai, China; 2 Department of Pathology, The Affiliated Yantai Yuhuangding Hospital, Qingdao University, Yantai, China; 3 Biomedical Research Center, Zhongshan Hospital, Fudan University, Shanghai, China; 4 Department of Pulmonary Medicine, Huashan Hospital, Fudan University, Shanghai, China; 5 State Key Laboratory of Respiratory Disease, Guangzhou Medical University, Guangzhou, China; University of South Alabama Mitchell Cancer Institute, UNITED STATES

## Abstract

**Background and objective:**

Chemotherapy drugs, such as cisplatin (DDP), improve the survival of patients with lung cancer by inducing apoptosis in cancer cells, which quickly develop resistance to DDP through uncharacterized mechanisms. Glioma Pathogenesis-Related Protein 1 (GLIPR1) plays an important role in cell proliferation, migration and apoptosis. However, the expression and function of GLIPR1 in mediating DDP resistance in human lung adenocarcinoma A549/DDP and human large cell lung cancer H460/DDP cells has not yet been reported.

**Methods:**

In this study, real-time PCR (RT-PCR) and western blot were used to examine the mRNA and protein expression of GLIPR1, respectively. Bright-field microscopy, the cell counting kit-8 (CCK-8) assay, flow cytometry analysis and JC-1 dye were used to measure the cellular morphology, proliferation, apoptosis and mitochondrial membrane potential, respectively.

**Results:**

Compared to human lung adenocarcinoma A549 cells, the mRNA and protein expression of GLIPR1 were significantly increased in DDP-resistant A549/DDP cells (p < 0.05). Similarly, the mRNA level of GLIPR1 in DDP-resistant H460/DDP cells was also significantly higher than that in DDP-sensitive H460 cells (p < 0.05). Silencing of GLIPR1 in A549/DDP and H460/DDP cells led to increased apoptosis via a mitochondrial signaling pathway following incubation with various concentrations of DDP. Furthermore, GLIPR1 downregulation markedly reduced the protein expression of Bcl-2, and increased the cleaved Poly (ADP-Ribose) Polymerase (PARP) and cleaved caspase-3 in DDP-resistant A549/DDP cells.

**Conclusion:**

In this study, we demonstrated for the first time that GLIPR1 could modulate the response of DDP-resistant A549/DDP and H460/DDP cells to cisplatin. Therefore, GLIPR1 deserves further investigation in the context of none-small lung cancer (NSCLC).

## Introduction

The highest incidence of malignant tumors throughout the world is attributable to lung cancer [1]. More than 2.2 million patients are diagnosed with lung cancer every year, and a large number of them are diagnosed at advanced stages [2]. Chemotherapy improves the survival of both patients with early stage cancer after surgery and patients with advanced non-small cell lung cancer (NSCLC) [3–4]. Cytotoxic drugs, such as cisplatin (DDP), could induce DNA damage through various signaling molecules, such as B-cell lymphoma 2 (Bcl-2) and c-Jun N-terminal kinase (JNK) [5–6]. Although lung cancer cells quickly develop resistance to DDP, the underlying molecular mechanism of this resistance has not been fully characterized [7].

Glioma Pathogenesis-Related Protein 1 (GLIPR1), a p53 targeting gene, was originally identified as a tumor suppressor in prostate cancer [8–10]. The expression of GLIPR1 was reduced in prostate and lung cancer cells compared to normal cells [9, 11]. Additionally, overexpression of GLIPR1 induced apoptosis of lung cancer cells [11] and prostate cancer cells by activating reactive oxygen species/the JNK pathway [12], downregulating c-Myc [13], or suppressing AURKA and TPX2 [14]. In contrast, GLIPR1 is overexpressed in astrocytic [15–19], wilms [20], acute myeloid leukemia [21], and melanoma [22] cancers. The overexpression of GLIPR1 increases glioma cell proliferation [18–19, 23], whereas the downregulation of GLIPR1 decreases the proliferation of glioma [18, 23] and melanoma [22] cells. However, the role of GLIPR1 in mediating DDP resistance in human lung adenocarcinoma A549/DDP and human large cell lung cancer H460/DDP cells has not yet been reported.

In this study, we found that the mRNA and protein expression of GLIPR1 were significantly increased in DDP-resistant A549/DDP cells compared to DDP-sensitive A549 cells (p < 0.05). The mRNA level of GLIPR1 in DDP-resistant H460/DDP cells was also significantly higher than that in DDP-sensitive H460 cells (p < 0.05). Silencing of GLIPR1 in A549/DDP and H460/DDP cells led to increased apoptosis via a mitochondrial signaling pathway following incubation with various concentrations of DDP. Furthermore, GLIPR1 downregulation significantly increased the presence of activated caspase-3 and cleaved Poly (ADP-Ribose) Polymerase (PARP), and markedly reduced the protein expression of Bcl-2, which is highly expressed in A549/DDP cells and plays a critical role in the DDP resistance of A549/DDP cells [6].

## Materials and methods

### Cell culture

The human lung adenocarcinoma cell line A549 and the DDP-resistant cell line A549/DDP were purchased from the Xiangya Cell Center, Central South China University (Changsha, China). The human large cell lung cancer cell line H460 was obtained from the American Type Culture Collection (ATCC). The DDP-resistant cell line H460/DDP was generated by treating the cells with sequentially increased cisplatin [24]. The cells were cultured in RPMI 1640 medium (Invitrogen, Carlsbad, CA, USA) supplemented with 10% heat-inactivated fetal bovine serum and 100 U/ml penicillin/streptomycin. The DDP resistance of A549/DDP and H460/DDP was maintained by adding 2 g/ml DDP (Sigma-Aldrich, St. Louis, USA). The cells were grown as monolayers in a humidified atmosphere containing 5% CO2 at 37°C.

### Lentiviral construction and infection

Short hairpin RNA (shRNA) vectors against the GLIPR1 genes shG-1 (TRCN0000123176) and shG-2 (TRCN0000123178) were obtained from TRC (The RNAi Consortium). Lentiviral plasmids containing GV298-shG-1, -shG-2, and -negative were obtained from GeneChem (Shanghai, China). Lentiviral particles were produced by the transfection of HEK 293T cells with the lentiviral plasmids. For viral infection, A549/DDP and H460/DDP cells were plated in 6-well plates (1×10^5^ cells/well), grown to 50–70% confluence, and incubated with medium containing virus and 4 μg/mL polybrene for 16 hours at a multiplicity of infection (MOI) of 20.

### Cell viability

The Cell Counting Kit-8 (CCK-8; Dojindo Laboratories, Japan) was used to assess the rate of cell proliferation. In brief, transfected A549/DDP and H460/DDP cells were plated in 96-well plates at approximately 2000 cells per well with 200 μL of culture medium and were treated with DDP at different concentrations. After 24 hours, 10 μl of CCK8 solution was applied to each well, and the plates were incubated for 1 h at 37°C. Finally, the absorbance values at 450 nm were determined using a microplate reader (Multiskan, Thermo, USA) with a reference wavelength of 650 nm. All of the experiments were conducted at least in triplicate.

### EdU incorporation assay

The cells were incubated with 10 μM EdU (5-ethynyl-2’-deoxyuridine, Invitrogen) for 4 hours and then fixed with 3.7% formaldehyde in PBS for 15 minutes at room temperature. The EdU was detected for EdU incorporation according to manufacturer’s recommendations. Confocal imaging was performed on a Nikon A1R confocal laser scanning microscope system (Nikon Corp., Tokyo, Japan). A549/DDP cells positive for EdU incorporation and positive for Hoechst 33342 staining were counted by using ImageJ (v. 1.42, Wayne Rasband, NIH), and used to calculate the percentage of EdU-positive cells.

### Detecting apoptosis by flow cytometry

An annexin V-FITC and propidium iodide (PI) double staining kit (Invitrogen, Carlsbad, CA, USA) was used to analyze cellular apoptosis. Transfected A549/DDP and H460/DDP cells were seeded in 6-well plates (5×10^5^ cells/well) and treated with DDP at different concentrations. After 24 hours, the cells were digested with trypsin (Gibco^®^ Trypsin-EDTA, Invitrogen, Carlsbad, CA, USA), washed with PBS three times, suspended in 500 μl of binding buffer, and then incubated with 5 μl of FITC-conjugated Annexin-V and 5 μl of PI for 15 min at room temperature in the dark. The stained cells were detected using the BD FACS Aria II flow cytometer (BD biosciences, San Jose, California, USA).

### Mitochondrial membrane potential measurement

The MitoProbe^™^ JC-1 assay kit (Thermo Fisher Scientific Inc., MA, USA) was used to detect changes in mitochondrial membrane potential. The assay was performed according to the manufacturer’s instructions, and the results of the assay were obtained by the BD FACS Aria II flow cytometer. JC-1 forms J-aggregates emitting red fluorescence at 590 nm in healthy mitochondria and J-monomers emitting green fluorescence at 490 nm in depolarized mitochondria. An increased ratio of J-monomers indicates mitochondrial damage. Carbonyl cyanide m-chlorophenylhydrazone (CCCP, 50 μM), a mitochondrial membrane potential disruptor, was used as a positive control.

### Quantitative RT-PCR

Total RNA was extracted using TRIzol reagent (Invitrogen, USA), and cDNA was synthesized using reverse transcriptase (TOYOBO, Japan). The RNA (1%) was reverse transcribed to complementary deoxyribonucleic acid, and 20 ng of complementary DNA was used as the template for RT-PCR. The amplification cycling reactions (40 cycles) were performed as follows: 15 seconds at 95°C, 15 seconds at 60°C and 45 seconds at 72°C. The primer sequences included the following:

GLIPR1 sense 5’- CCGCCATCACAAACTGGTAT-3’,

GLIPR1anti-sense 5’- TCTGCCCAAACAACCTGAGT-3’.

β-actin sense 5’- CTGGCACCCAGCACAATG -3’,

β-actin anti-sense 5’- CCGATCCACACGGAGTACTTG -3’.

Gene expression was normalized to β-actin and was measured by 2^-ΔΔCT^. RT-PCR was performed at least 3 separate times in triplicate.

### Western blot assay

Total protein was extracted using a RIPA kit (Beyotime Biotechnology Inc., Nantong, China), separated on polyacrylamide gels, and transferred to PVDF membranes. The membranes were incubated with anti-GLIPR1 (Abcam, Cambridge, MA, USA), cleaved caspase-3 (Asp175) [Cell Signaling Technology (CST), MA, USA], cleaved PARP (Asp214) (CST), anti-Bcl-2(CST), and anti-actin (CST) at 4°C overnight and were then incubated with horseradish peroxidase-conjugated goat anti-rabbit or anti-mouse immunoglobulin G at room temperature for 1 hour. The proteins were visualized using Pierce ECL western blotting substrate and autoradiography. The blots were analyzed using Quantity One 4.6.

### Intracellular signaling array

Cell extracts were prepared and analyzed using the PathScan intracellular signaling array kit (Catalog no. 7323S; Cell Signaling Technology) and PathScan stress and apoptosis signaling antibody array kit (Catalog no. 12856S; Cell Signaling Technology) according to the manufacturer's instruction. The PathScan Intracellular Signaling Array Kit could simultaneously detect eighteen phosphorylated or cleaved intracellular signaling molecules including ERK1/2, mammalian target or rapamycin (mTOR), mitogen-activated protein kinase (MAPK), B-cell lymphoma-2-associated death domain (Bad). The PathScan Stress and Apoptosis Signaling Antibody Array could simultaneously detect nineteen apoptosis related signaling molecules including cleaved caspases (caspase 3 and 9) and PARP.

### Methylation analysis

Genomic DNA from A549 and A549/DDP cells was isolated using DNeasy Blood & Tissue Kit (Qiagen, Valencia, CA, USA). Bisulfite conversion of genomic DNA was performed using the EpiTect Bisulfite Kit (Qiagen). The GLIPR1 promoter fragment was amplified by PCR and cloned into the Pmd18-T Vector (Takara, Japan). Five independent clones from each subject were sequenced for each of the amplified fragments. Primers are described as follows:

Forward: (from5’ to 3’) TGAAAATTATTGAAAAGATAGGG;

Reverse: (from 5’ to 3’) AAACCATCCAAACTATTATAACAA.

### Statistical analysis

The data were expressed as the means ± SD of at least three independent experiments. The statistical analysis was performed using one-way analysis of variance (ANOVA) followed by Bonferroni’s multiple comparison test. A p-value < 0.05 was considered statistically significant.

## Results

### GLIPR1 was upregulated in DDP-resistant A549/DDP and H460/DDP cells

**Fig 1:**
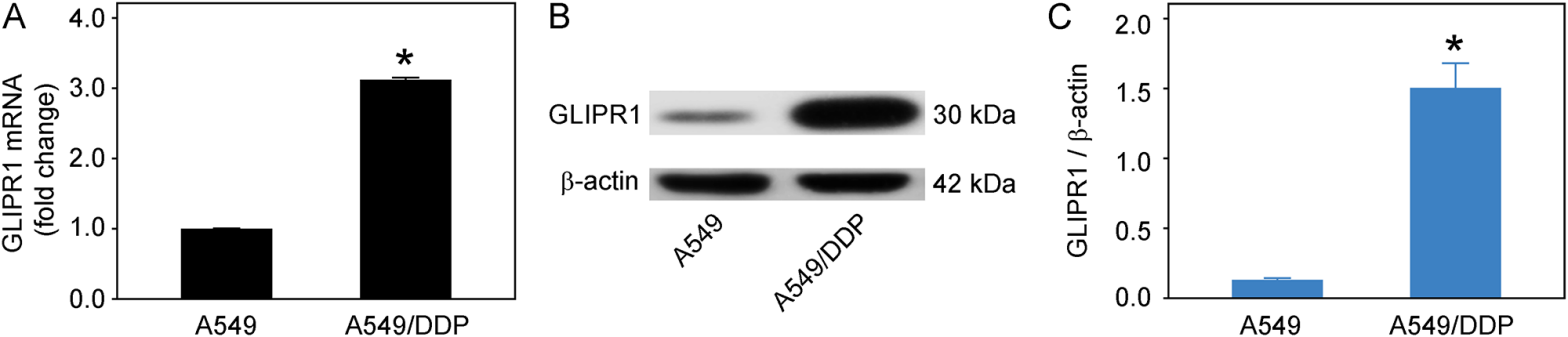
GLIPR1 was upregulated in DDP-resistant A549/DDP cells. A) The RT-PCR results showed that GLIPR1 mRNA was significantly increased in A549/DDP cells compared to A549 cells. The data were presented as the fold changes in gene expression normalized to β-actin and relative to A549 cells. B) Cellular GLIPR1 and β-actin were assessed by western blot. C) The statistical analysis demonstrated that the GLIPR1 protein was significantly upregulated in A549/DDP cells compared to A549 cells. The protein levels of GLIPR1 were normalized to β-actin. The data were representative of three similar experiments. * indicates a significant difference at p < 0.05 versus A549 cells.

To investigate the potential role of GLIPR1 in the development of chemotherapeutic drug resistance, we firstly compared the expression levels of GLIPR1 in DDP-sensitive and -resistant lung adenocarcinoma A549 cells. The RT-PCR results revealed that GLIPR1 mRNA was significantly increased in DDP-resistant A549/DDP cells compared to DDP-sensitive A549 cells (p < 0.05) (Fig 1A). Similarly, the western blot results showed elevated protein levels in A549/DDP cells compared to A549 cells, suggesting the role of GLIPR1 in chemoresistance (p < 0.05) (Fig 1B and 1C). To verify this, we generated DDP-resistant H460/DDP cells by treating the cells with sequentially increased cisplatin. The CCK-8 assay showed that the proliferation rate of DDP-resistant H460/DDP cells was significantly higher than that of DDP-sensitive H460 cells (p < 0.05) (S1A Fig). The RT-PCR results demonstrated that GLIPR1 mRNA is significantly increased in H460/DDP cells compared to H460 cells (p < 0.05) (S1B Fig).

### GLIPR1 mediated DDP resistance in A549/DDP and H460/DDP cells

**Fig 2:**
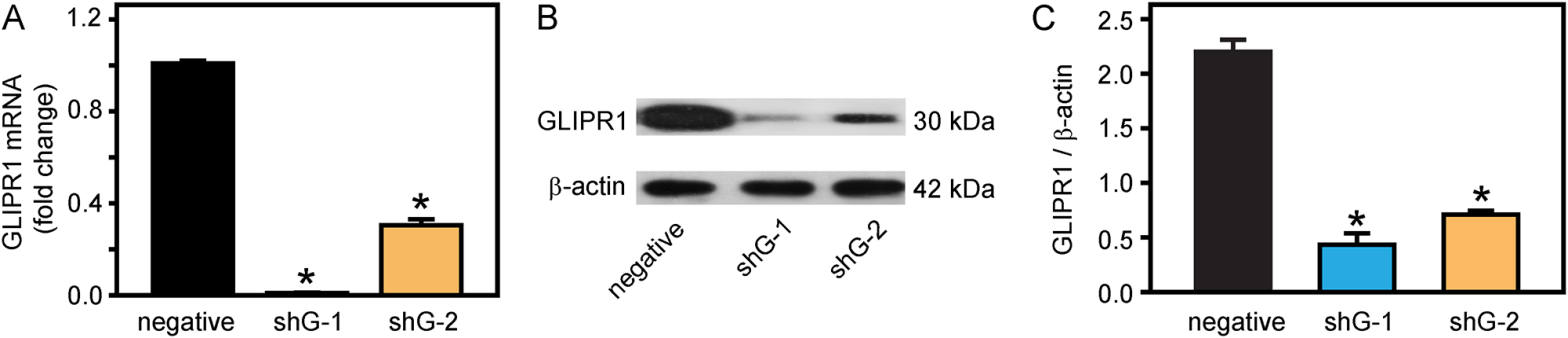
shRNA sequences targeting the GLIPR1 gene reduced GLIPR1 expression in A549/DDP cells. A) The RT-PCR results showed that shRNA sequences, shG-1 and shG-2, significantly reduced GLIPR1 mRNA expression in A549/DDP cells compared to the negative control. The data were presented as the fold changes in gene expression normalized to β-actin and relative to negative control. B) The western blot results showed that shG-1 and shG-2 reduced GLIPR1 protein in A549/DDP cells compared to the negative control. C) The statistical analysis demonstrated that shG-1 and shG-2 significantly downregulated GLIPR1 protein in A549/DDP cells compared to the negative control. The protein levels of GLIPR1 were normalized to β-actin. The data were representative of three similar experiments. The error bars represent mean values ± SD. * indicates a significant difference at p < 0.05 versus the negative control.

GLIPR1 shRNA or negative shRNA which could downregulate GLIPR1 expression in DDP-resistant A549/DDP cells were stably transfected into A549/DDP cells. The RT-PCR results demonstrated that both shG-1 and shG-2, two shRNA sequences targeting the GLIPR1 gene, could significantly reduce GLIPR1 mRNA expression in A549/DDP cells (p < 0.05) (Fig 2A). As confirmation, the western blot results revealed that shG-1 and shG-2 both significantly decreased the GLIPR1 protein levels in A549/DDP cells (p < 0.05) (Fig 2B and 2C).

**Fig 3:**
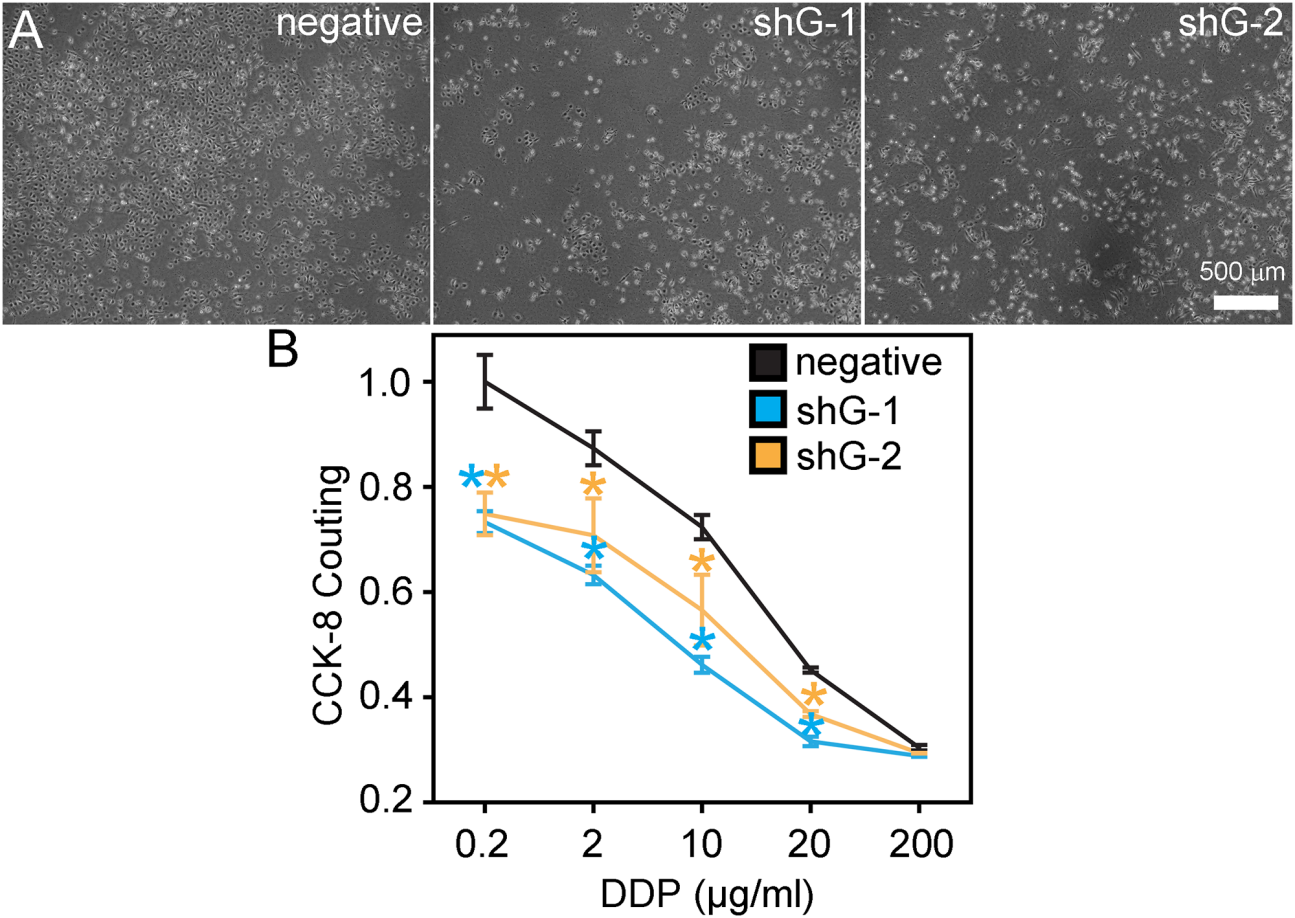
GLIPR1 mediated DDP resistance in A549/DDP cells. A) Bright-field images of A549/DDP cells 120 hours after transfection with GLIPR1 shRNA or negative shRNA incubated with 2 μg/ml DDP induced cell morphological changes. B) Cell proliferation and the viability of A549/DDP cells transfected with GLIPR1 shRNA or negative shRNA incubated with 0.2, 2, 10, 20, and 200 μg/ml DDP were measured by CCK-8. One representative experiment with n = 3 is shown. The error bars represent mean values ± SD. * indicates a significant difference at p < 0.05 versus the negative control.

**Fig 4:**
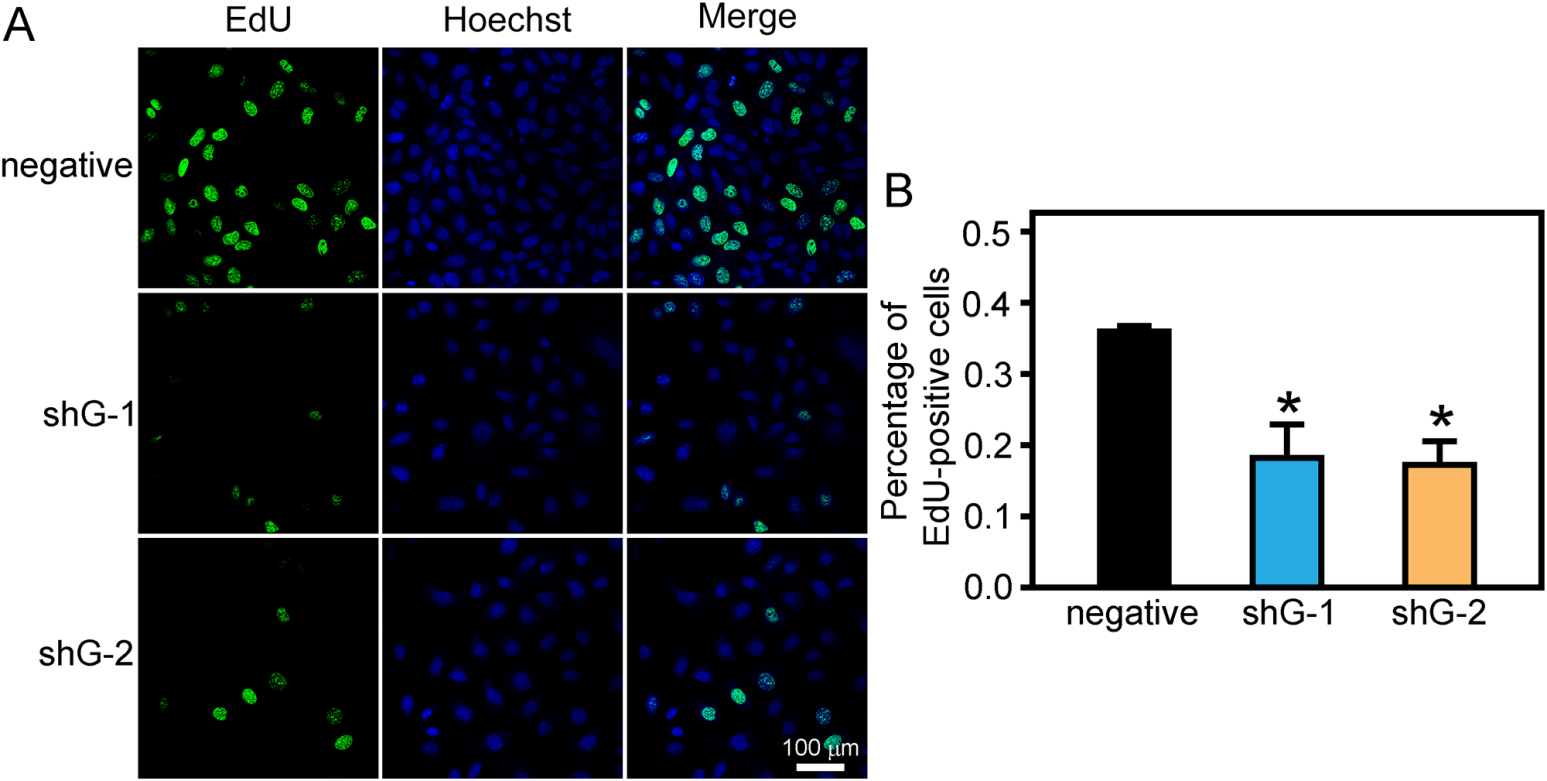
Silencing GLIPR1 decreased the proliferation of A549/DDP cells. A) A549/DDP cells transfected with with GLIPR1 shRNA or negative shRNA were treated with 2 μg/ml DDP, stained with EdU and Hoechst 33342. B) the percentage of EdU-positive cells in negative control group was significantly higher than those in shG-1 and shG-2 groups. The data were representative of at least three similar experiments. * indicates a significant difference at p < 0.05 versus the negative control.

To study the effect of GLIPR1 downregulation on the apoptosis of A549/DDP cells, bright-field images of GLIPR1 shRNA or negative shRNA stably transfected A549/DDP cells were collected 120 hours after transfection. Morphological examination of A549/DDP cells demonstrated that shG-1 and shG-2 resulted in decreased cell proliferation (Fig 3A). To investigate if GLIPR1 could mediate DDP resistance in A549/DDP cells, we analyzed the effect of shRNA-induced GLIPR1 downregulation on the proliferation of A549/DDP cells following DDP treatment. The CCK-8 assay showed that shG-1 and shG-2 significantly inhibited the growth of A549/DDP cells compared to the negative control after treatment with 0.2, 2, 10, 20 μg/ml DDP (p < 0.05) (Fig 3B). In addition, the effects of silencing GLIPR1 on the proliferation of A549/DDP cells following DDP treatment was measured by using EdU incorporation. The results showed that there were about 37% EdU positive cells in negative control group, and about 20% in shG-1 and shG-2 groups when incubated with 2 μg/ml DDP (Fig 4A and 4B), suggesting silencing GLIPR1 significantly reduced the proliferation of A549/DDP cells following DDP treatment.

**Fig 5:**
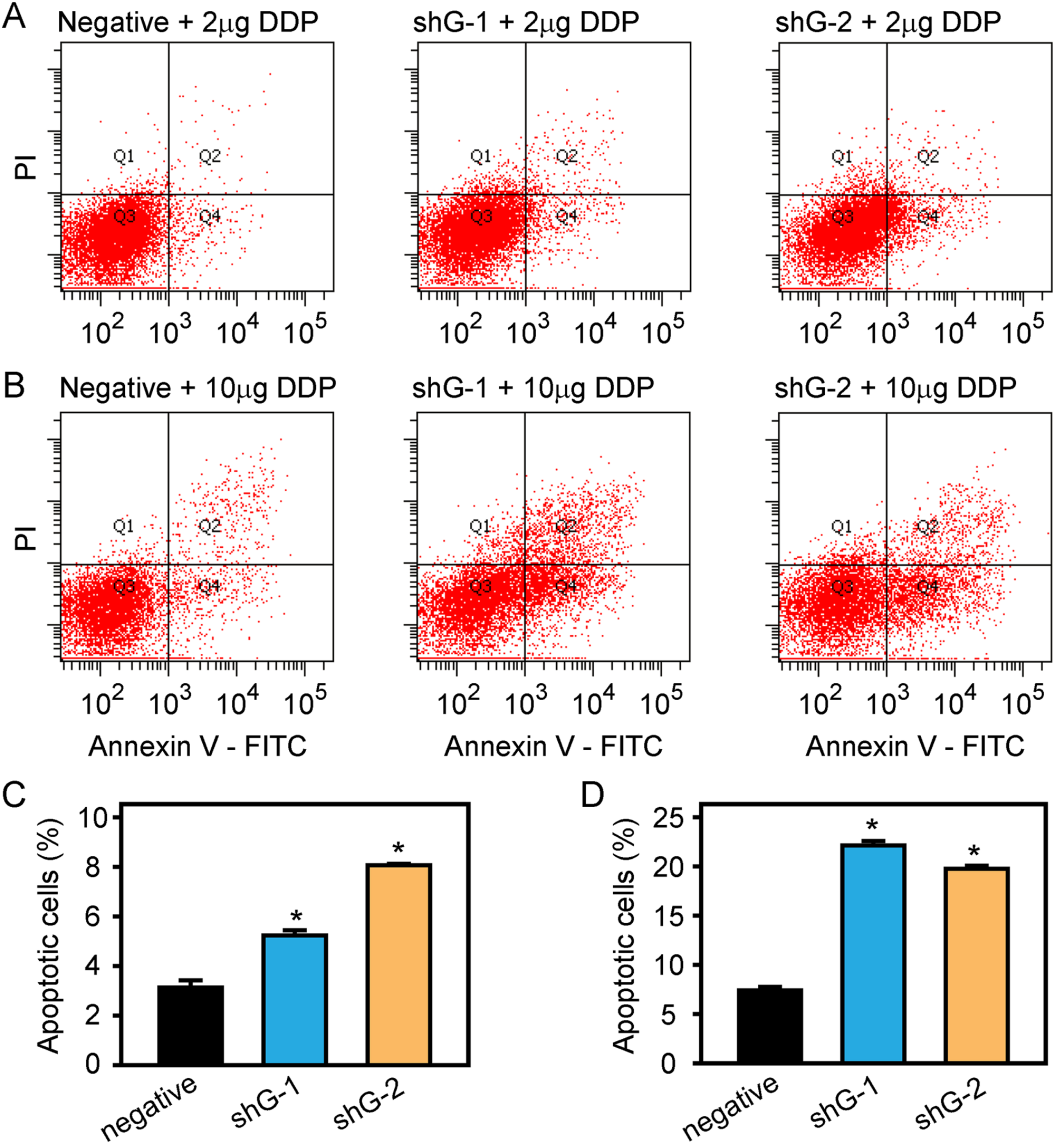
Flow cytometric analysis of apoptosis induction in A549/DDP cells following DDP treatment. A549/DDP cells transfected with GLIPR1 shRNA or negative shRNA were treated with 2 μg/ml DDP (A) or 10 μg/ml DDP (B) for 24 hours, stained with FITC-annexin V/PI, and then analyzed by flow cytometry. The statistical analysis revealed that shG-1 or shG-2 significantly increased the apoptosis of A549/DDP cells compared to the negative control incubated with 2 μg/ml (C) or 10 μg/ml DDP (D). The data were representative of three similar experiments. * indicates a significant difference at p < 0.05 versus the negative control.

Moreover, an annexin V-FITC/PI double staining assay and flow cytometry analysis were performed. The cells in the upper-right (UR, Q2) and lower-right (LR, Q4) quadrants of the FACS histogram represent apoptotic cells. As shown in Fig 5, the apoptosis rates of A549/DDP cells transfected with shG-1 or shG-2 were significantly increased compared to that of the negative control when incubated with 2 μg/ml DDP (p < 0.05) (Fig 5A and 5C), with 10 μg/ml DDP for 24 hours (p < 0.05) (Fig 5B and 5D), or in the absence of DDP (S2A and S2B Fig).

Furthermore, GLIPR1 shRNA or negative shRNA were stably transfected into H460/DDP cells to investigate the effect of GLIPR1 downregulation on the apoptosis of H460/DDP cells. The RT-PCR results demonstrated that both shG-1 and shG-2 could significantly reduce GLIPR1 mRNA expression in H460/DDP cells (p < 0.05) (S1C Fig). The CCK-8 assay showed that shG-1 and shG-2 significantly inhibited the growth of H460/DDP cells compared to the negative control after treatment with 0, 2, 10 μg/ml DDP (S1D Fig). The annexin V-FITC/PI double staining assay and flow cytometry analysis showed that the apoptosis rates of H460/DDP cells transfected with shG-1 or shG-2 were significantly increased compared to that of the negative control when incubated with 2 μg/ml DDP for 24 hours (S1E and S1F Fig).

### Downregulation of GLIPR1 decreased mitochondrial membrane potential

**Fig 6:**
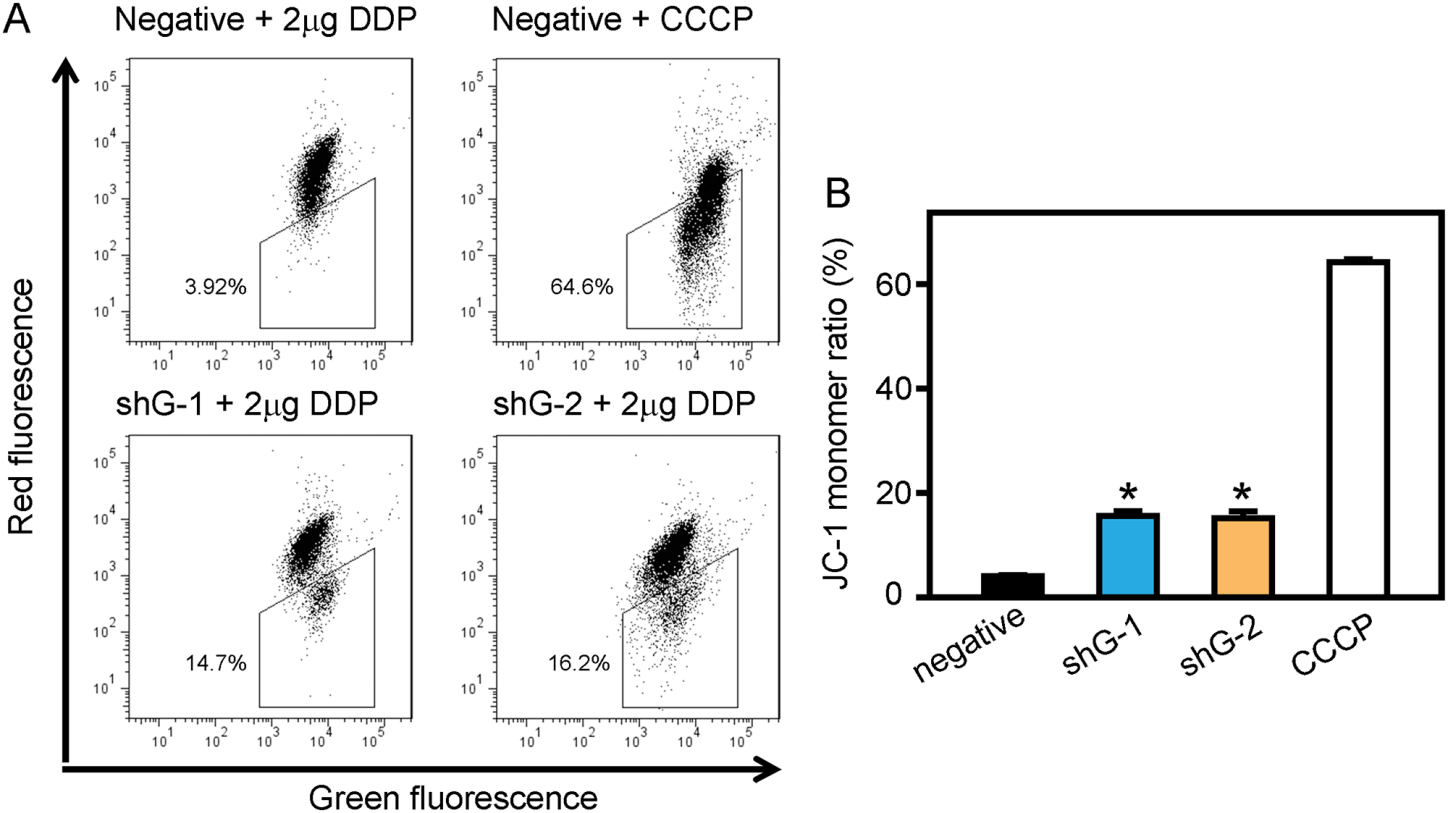
Downregulation of GLIPR1 decreases mitochondrial membrane potential. A) Representative histograms showing flow cytometry analysis of JC-1 staining. CCCP was used as a positive control. B) The statistical analysis revealed that shG-1 or shG-2 significantly increased the JC-1 monomer ratio of A549/DDP cells compared to the negative control incubated with 2 μg/ml DDP. The data are representative of three similar experiments. * indicates a significant difference at p < 0.05 versus the negative control.

Depolarization of the mitochondrial membrane potential is an indicator of the cell apoptosis [25]. In this study, we stained GLIPR1 shRNA or negative shRNA stably transfected A549/DDP cells with JC-1, which accumulates in healthy mitochondria as J-aggregates emitting red fluorescence, while in depolarized or damaged mitochondria as JC-1 monomers emitting green fluorescence. We found that the JC-1 monomer ratio of A549/DDP cells transfected with shG-1 or shG-2 were significantly increased compared to that of the negative control when incubated with 2 μg/ml DDP (Fig 6A and 6B), or in the absence of DDP (S2C and S2D Fig).

### GLIPR1 regulated Bcl-2, cleaved caspase-3, and cleaved PARP

**Fig 7:**
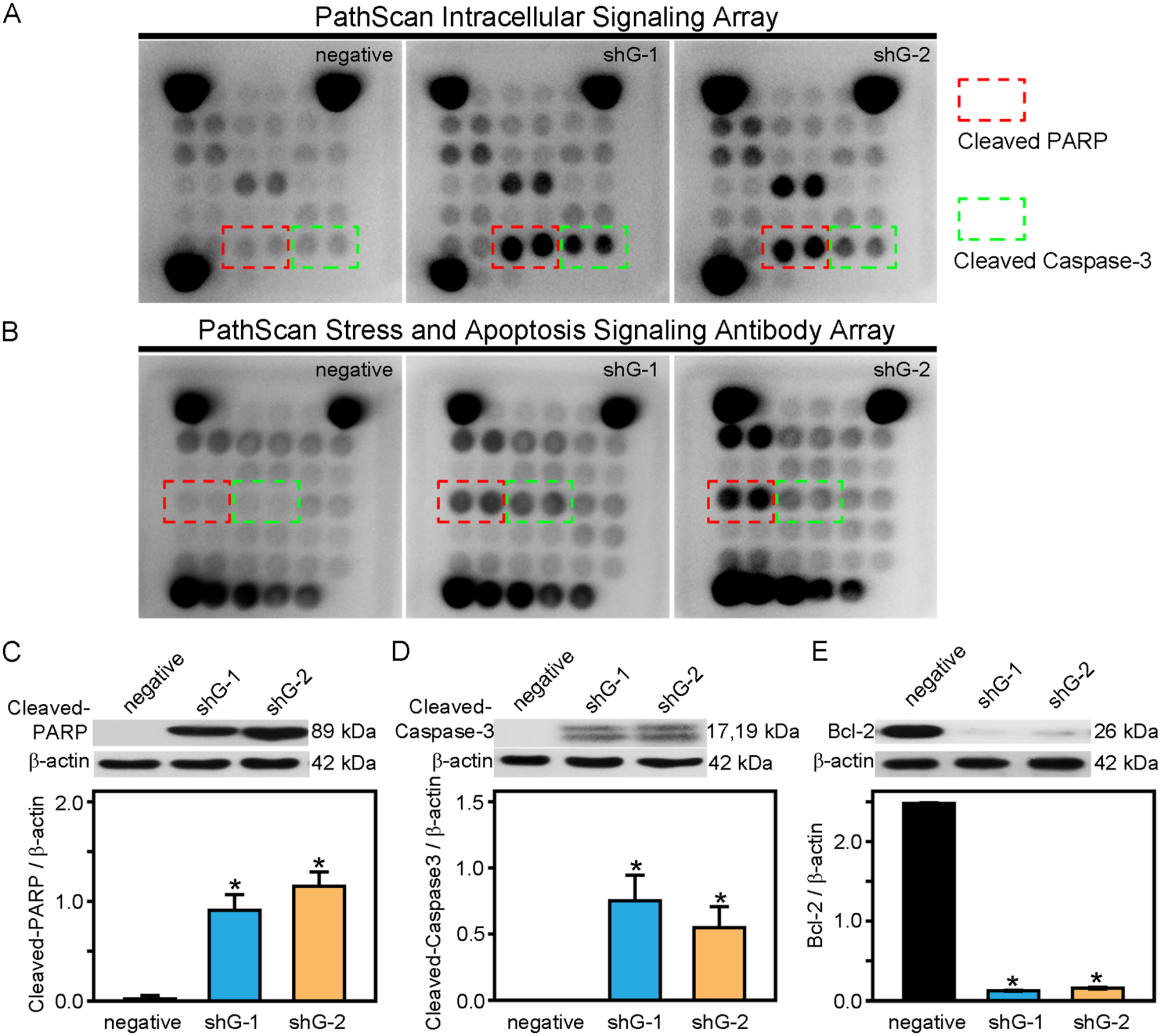
GLIPR1 regulates Bcl-2, cleaved caspase-3, and cleaved PARP. A) Representative chemiluminescent images produced using the PathScan intracellular signaling array kit and the PathScan stress and apoptosis signaling antibody array kit (B). Red box: cleaved PARP; Green box: cleaved caspase-3. C) The upper panel showed that the levels of cleaved PARP were increased in A549/DDP cells transfected with GLIPR1 shRNA. The lower panel demonstrated that shG-1 or shG-2 significantly increased the levels of cleaved PARP compared to the negative control. The figures are representative profiles of at least three experiments. * indicates a significant difference at p < 0.05 versus the negative control. D) The upper panel showed that the levels of cleaved caspase-3 were increased in A549/DDP cells transfected with GLIPR1 shRNA. The lower panel demonstrated that shG-1 or shG-2 significantly increased the levels of cleaved caspase-3 compared to the negative control. E) The upper panel showed that the expression of Bcl-2 protein were increased in A549/DDP cells transfected with GLIPR1 shRNA. The lower panel demonstrated that shG-1 or shG-2 significantly increased the expression of Bcl-2 protein compared to the negative control.

The molecular mechanism underlying GLIPR1 mediated DDP resistance in A549/DDP cells was investigated by examined the phosphorylation or activation of eighteen signaling molecules using the PathScan intracellular signaling array kit. It was found that the levels of cleaved PARP (Asp214), a DNA repair enzyme, and cleaved caspase-3 (Asp175), a pro-apoptotic protein, in A549/DDP cells transfected with shG-1 or shG-2 were markedly increased compared with that in negative control cells (Fig 7A). Since GLIPR1 mediates the apoptosis-associated proteins in A549/DDP cells, the PathScan stress and apoptosis signaling antibody array kit was then used to monitor the nineteen apoptosis related signaling molecules. It was confirmed that there was a significant increase in the expression levels of cleaved PARP and cleaved caspase-3 in A549/DDP cells transfected with shG-1 or shG-2 (Fig 7B). Western blot results further confirmed that cleaved PARP and cleaved caspase-3 were significantly increased when GLIPR1 was downregulated in A549/DDP cells (p < 0.05) (Fig 7C and 7D).

The anti-apoptotic protein Bcl-2 plays an important role in sensitizing DDP-resistant A549/DDP cells to DDP [6] and is a upstream signaling molecule of cleaved caspase-3 and PARP [26]. We then explored the effect of GLIPR1 reduction on the expression of the Bcl-2 protein. The western blot results clearly showed that shG-1 and shG-2 markedly reduced the protein expression of Bcl-2 in A549/DDP cells compared to the negative control (p < 0.05) (Fig 7E).

### Methylation was not conjunction with the high expression of GLIPR1 in A549/DDP cells

**Fig 8:**
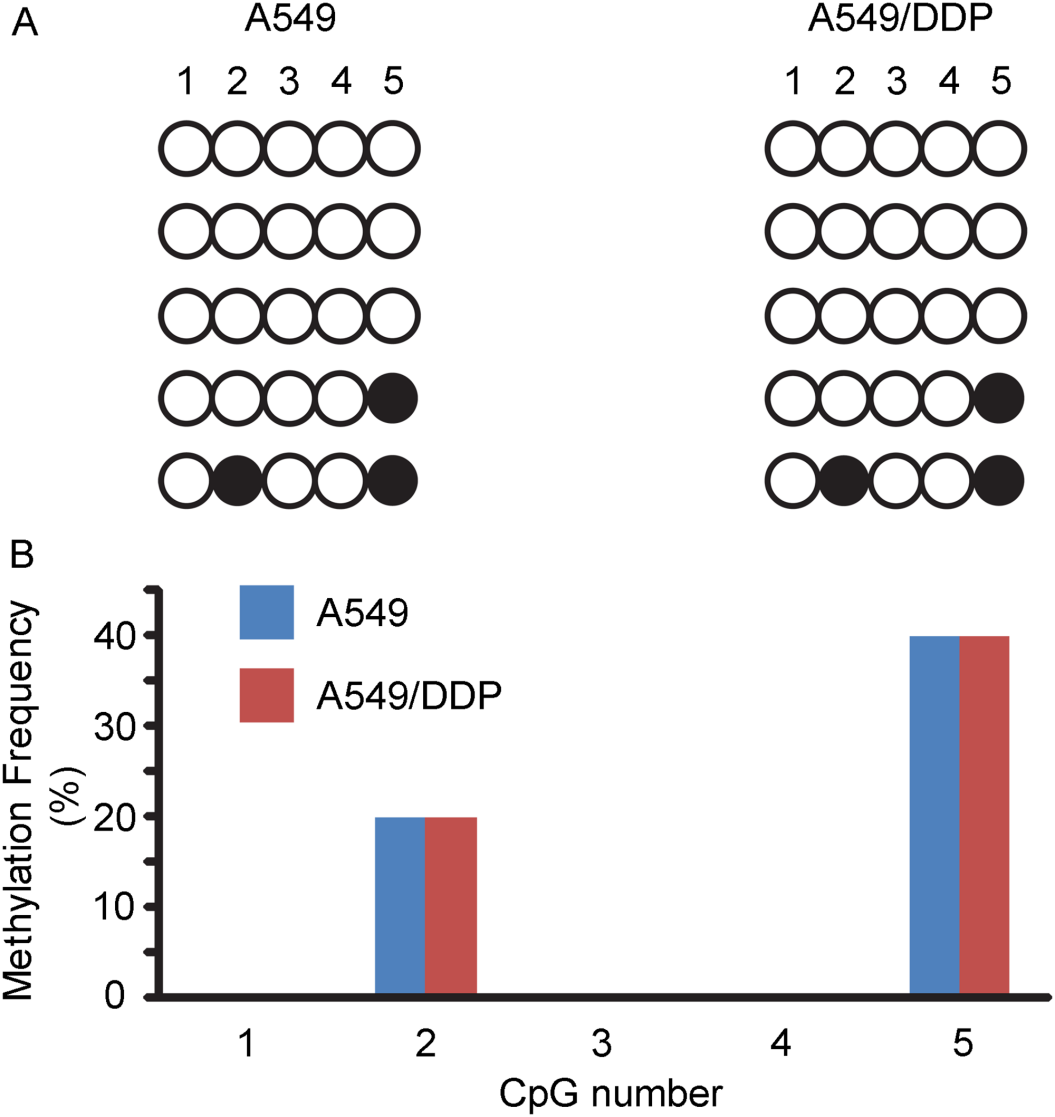
The epigenetic status of GLIPR1 in DDP-sensitive and -resistant human lung adenocarcinoma A549 cells. A) Each row shows the methylation status for 5 single DNA clones (numbered # 1 to 5). The filled circle represents the methylated CpG, and the empty circle the unmethylated CpG. B) Methylation frequency at each of the 5 CpG sites in the GLIPR1 promoter in A549 and A549/DDP cells.

To examine whether the epigenetic status of GLIPR1 in DDP-sensitive and -resistant human lung adenocarcinoma A549/DDP cells was responsible for the high expression of GLIPR1 in A549/DDP cells [9, 15, 20], bisulfite sequencing primers were designed to amplify a 264 bp region 5′ of the transcription start site containing five CpG sites as previously reported.[15] GLIPR1 bisulfite sequencing data were obtained on a minimum of 5 clones prepared from each of both A549 and A549/DDP cells (Fig 8A). The GLIPR1 promoter sequences amplified from A549/DDP cells showed no differences in methylation as compared to A549 cells (Fig 8B).

## Discussion

Drug resistance is one of the primary causative agents of poor prognoses in patients with advanced NSCLC. Thus, it is necessary to identify novel pharmaceutical targets in the treatment of patients with resistance to DDP. In this study, we examined the expression of GLIPR1 in DDP-sensitive and -resistant human lung adenocarcinoma A549 and human large cell lung cancer H460 cells and the role of GLIPR1 in mediating the resistant of A549/DDP and H460/DDP cells to DDP. GLIPR1 has been extensively studied in various cancers, including prostate [8–9, 12–14], astrocytic [18], wilms [20], acute myeloid leukemia [21], melanoma [22], and lung cancers [11]. However, its expression and function in DDP-resistant A549/DDP and H460/DDP cells have not yet been investigated.

In the present study, the results showed that compared with human lung adenocarcinoma A549 cells, the mRNA and protein expression of GLIPR1 were significantly increased in DDP-resistant A549/DDP cells. Similarly, the mRNA level of GLIPR1 in DDP-resistant H460/DDP cells was also significantly higher than that in DDP-sensitive H460 cells. GLIPR1 has been found to be expressed at high levels in astrocytic [15–19, 23], wilms [20], acute myeloid leukemia [21], and melanoma [22] cancers and at low levels in prostate [8–10], and lung cancer [11]. The differential expression pattern is possibly due to the epigenetic status of GLIPR1 in different cancers. It has been found that DNA hypomethylation of the GLIPR1 gene promoter led to its overexpression in glioma [15] and wilms [20] tumors, whereas hypermethylation of the GLIPR1 gene promoter led to the downregulation of its mRNA expression in prostate cancer [9]. Thus, the epigenetic status of GLIPR1 in DDP-sensitive and -resistant human lung adenocarcinoma A549 cells was examined; however, there were no differences in the levels of methylation between A549 and A549/DDP cells. It is possible that other mechanisms might be involved in the increased expression of GLIPR1 in A549/DDP cells.

The function of GLIPR1 remains controversial. The overexpression of GLIPR1 has been demonstrated to induce apoptosis in prostate cancer cells [9, 12–14]; however, overexpression of GLIPR1 increased glioma cell proliferation [18–19, 23] and downregulation of GLIPR1 decreased the proliferation of glioma [18, 23] and melanoma [22] cells. Thus, it is necessary to investigate the role of GLIPR1 in mediating the A549/DDP and H460/DDP cell response to DDP. In the study, we found that, in the absence of DDP, GLIPR1 downregulation resulted in slightly increased cellular apoptosis and decreased mitochondrial membrane potential (S2 Fig); however, the percentage of cellular apoptosis and mitochondrial membrane potential decrease was more profound when incubated in DDP, especially in 10 μg/ml DDP. These results indicate that GLIPR1 is critical for cell survival and GLIPR1 downregulation sensitizes cells to DDP. Our results are consistent with studies in glioma and melanoma cells, in which GLIPR1 promotes cell proliferation.

Mitochondria play critical roles in the apoptosis of A549/DDP cells. In this study, GLIPR1 downregulation was found to cause mitochondrial damage as indicated by the loss of mitochondrial membrane potential. To further explore the mechanisms underlying GLIPR1-mediated DDP resistance in A549/DDP cells, PathScan intracellular signaling array kit and PathScan stress and apoptosis signaling antibody array kit were used to simultaneously detect various phosphorylated or cleaved intracellular signaling molecules. Finally, downregulation of GLIPR1 in A549/DDP cells was discovered significantly increased the cleaved PARP, a DNA repair enzyme, and cleaved caspase-3, a pro-apoptotic protein. Next, the protein expression of Bcl-2 in A549/DDP cells, an anti-apoptotic protein, was examined and found that the downregulation of GLIPR1 led to reduced protein levels of Bcl-2 in A549/DDP cells. Taken together, it was possible that downregulation of GLIPR1 induced the apoptosis of A549/DDP cells in a Bcl-2-dependent pathway and then activated caspase-3, following PARP cleavage.

Our results are consistent with previous studies in which the overexpression of GLIPR1 increased the expression of Bcl-2 [18], which was found to be highly expressed in A549/DDP cells, and the silencing of Bcl-2 induced an increase in cell apoptosis [6]. DDP is used as a first-line treatment among patients with advanced NSCLC; however, lung cancer cells quickly develop drug resistance. The results of this study suggest that A549/DDP cells increase DDP resistance by upregulating GLIPR1, which promotes cell proliferation by inducing Bcl-2 expression. Thus, our results revealed a novel signaling pathway mediating DDP resistance in A549/DDP cells. However, there are several biological limitation to the current study; for example, the animal models are needed to verify the cellular results.

### Conclusion

In summary, the mRNA and protein expression of GLIPR1 were significantly increased in DDP-resistant A549/DDP and H460/DDP cells. Silencing of GLIPR1 in A549/DDP cells induced apoptosis via a mitochondrial signaling pathway by decreasing the anti-apoptosis protein Bcl-2, and increasing cleaved caspase-3 and PARP. Our results suggest that GLIPR1 deserves further investigation in the context of NSCLC and further investigation is required to verify GLIPR1 has the potential to be a novel therapeutic target for DDP-resistant NSCLC patients.

## Supporting information

S1 Fig GLIPR1 mediates DDP resistance in H460/DDP cells. A) Cell proliferation and the viability of H460/DDP cells incubated with 0, 2, 6, and 10 μg/ml DDP was measured by CCK-8. One representative experiment with n = 3 is shown. The error bars represent mean values ± SD. * indicates a significant difference at p < 0.05 versus the DDP sensitive H460 cells. B) The RT-PCR results showed that GLIPR1 mRNA is significantly increased in H460/DDP cells compared to H460 cells. The data are presented as the fold changes in gene expression normalized to β-actin and relative to H460 cells. C) The RT-PCR results showed that shRNA sequences shG-1 and shG-2 significantly reduced GLIPR1 mRNA expression in H460/DDP cells compared to the negative control. The data are presented as the fold changes in gene expression normalized to β-actin and relative to negative control. The error bars represent mean values ± SD. * indicates a significant difference at p < 0.05 versus the negative control. D) Cell proliferation and the viability of H460/DDP cells transfected with GLIPR1 shRNA or negative shRNA incubated with 0, 2, and 10 μg/ml DDP was measured by CCK-8. One representative experiment with n = 3 is shown. The error bars represent mean values ± SD. * indicates a significant difference at p < 0.05 versus the negative control. E) H460/DDP cells transfected with GLIPR1 shRNA or negative shRNA were treated with 2 μg/ml DDP for 24 hours, stained with FITC-annexin V/PI, and then analyzed by flow cytometry. F) The statistical analysis revealed that shG-1 or shG-2 significantly increased the apoptosis of H460/DDP cells compared to the negative control. The data are representative of three similar experiments. * indicates a significant difference at p < 0.05 versus the negative control.
(TIF)

S2 Fig Downregulation of GLIPR1 decreases mitochondrial membrane potential and induces cellular apoptosis of H460/DDP cells. A) H460/DDP cells transfected with GLIPR1 shRNA or negative shRNA in the absence of DDP were stained with FITC-annexin V/PI, and then analyzed by flow cytometry. B) The statistical analysis revealed that shG-1 or shG-2 significantly increased the apoptosis of H460/DDP cells compared to the negative control. The data are representative of three similar experiments. * indicates a significant difference at p < 0.05 versus the negative control. C) Representative histograms showing flow cytometry analysis of JC-1 staining. D) The statistical analysis revealed that shG-1 or shG-2 significantly increased the JC-1 monomer ratio of H460/DDP cells compared to the negative control in the absence of DDP. The data are representative of three similar experiments. * indicates a significant difference at p < 0.05 versus the negative control.
(TIF)

## Abbreviations

ANOVA: analysis of variance
Bad: B-cell lymphoma-2-associated death domain
Bcl-2: B-cell lymphoma 2
CCK-8: cell counting kit-8
CST: Cell Signaling Technology
GLIPR1: Glioma Pathogenesis-Related Protein 1
JNK: c-Jun N-terminal kinase
MAPK: mitogen-activated protein kinase
mTOR: mammalian target or rapamycin
NSCLC: non-small cell lung cancer
PI: CCCP: propidium iodide; Carbonyl cyanide m-chlorophenylhydrazone
RT-PCR: real-time PCR
shRNA: Short hairpin RNA
