# Supplementary figures and images for: GLIPR1 modulates the response of cisplatin-resistant human lung cancer cells to cisplatin
Source: PLoS One. 2017 Aug 3;12(8):e0182410. doi: 10.1371/journal.pone.0182410 (PMC5542429; doi:10.1371/journal.pone.0182410)

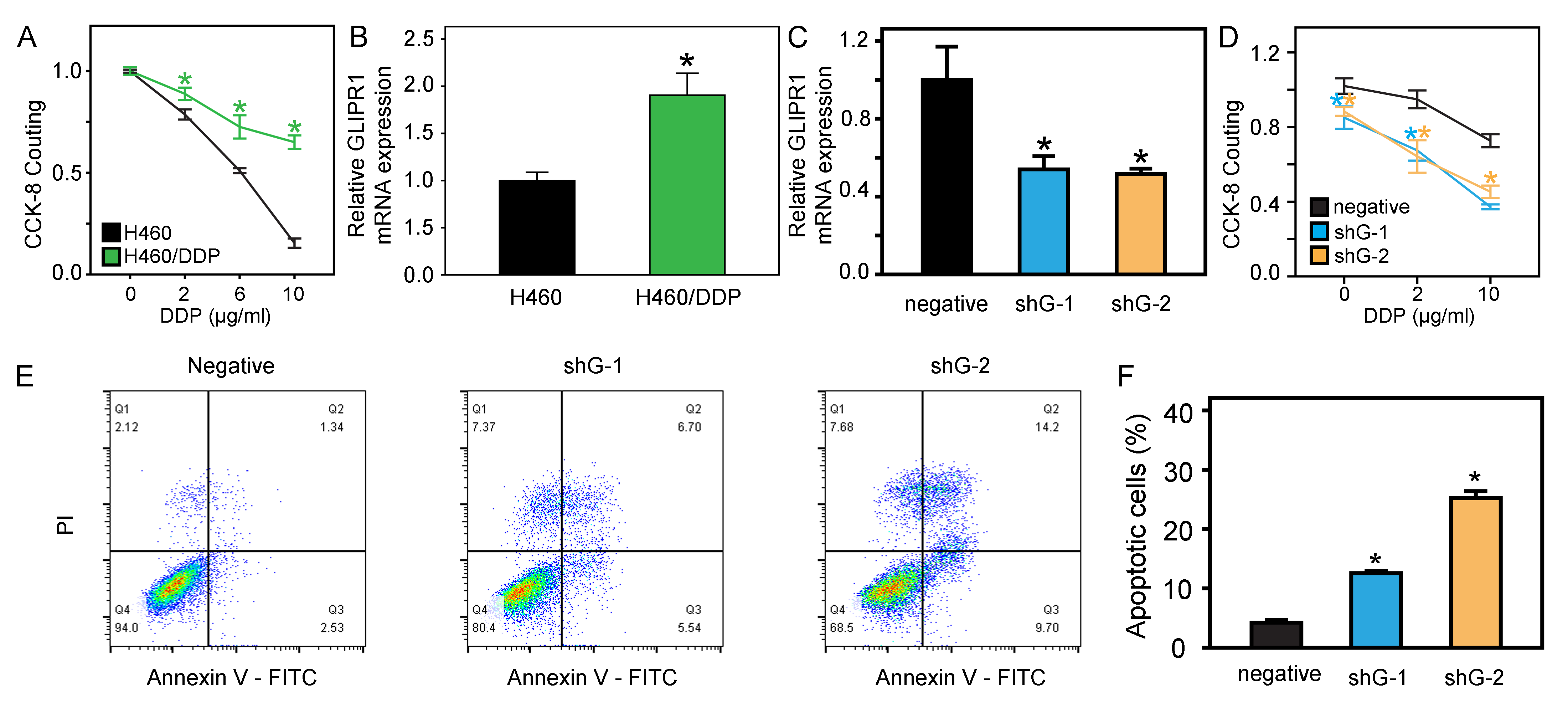

Supplement: S1 Fig — A) Cell proliferation and the viability of H460/DDP cells incubated with 0, 2, 6, and 10 μg/ml DDP was measured by CCK-8. One representative experiment with n = 3 is shown. The error bars represent mean values ± SD. * indicates a significant difference at p < 0.05 versus the DDP sensitive H460 cells. B) The RT-PCR results showed that GLIPR1 mRNA is significantly increased in H460/DDP cells compared to H460 cells. The data are presented as the fold changes in gene expression normalized to β-actin and relative to H460 cells. C) The RT-PCR results showed that shRNA sequences shG-1 and shG-2 significantly reduced GLIPR1 mRNA expression in H460/DDP cells compared to the negative control. The data are presented as the fold changes in gene expression normalized to β-actin and relative to negative control. The error bars represent mean values ± SD. * indicates a significant difference at p < 0.05 versus the negative control. D) Cell proliferation and the viability of H460/DDP cells transfected with GLIPR1 shRNA or negative shRNA incubated with 0, 2, and 10 μg/ml DDP was measured by CCK-8. One representative experiment with n = 3 is shown. The error bars represent mean values ± SD. * indicates a significant difference at p < 0.05 versus the negative control. E) H460/DDP cells transfected with GLIPR1 shRNA or negative shRNA were treated with 2 μg/ml DDP for 24 hours, stained with FITC-annexin V/PI, and then analyzed by flow cytometry. F) The statistical analysis revealed that shG-1 or shG-2 significantly increased the apoptosis of H460/DDP cells compared to the negative control. The data are representative of three similar experiments. * indicates a significant difference at p < 0.05 versus the negative control. (TIF) [file pone.0182410.s001.tif]

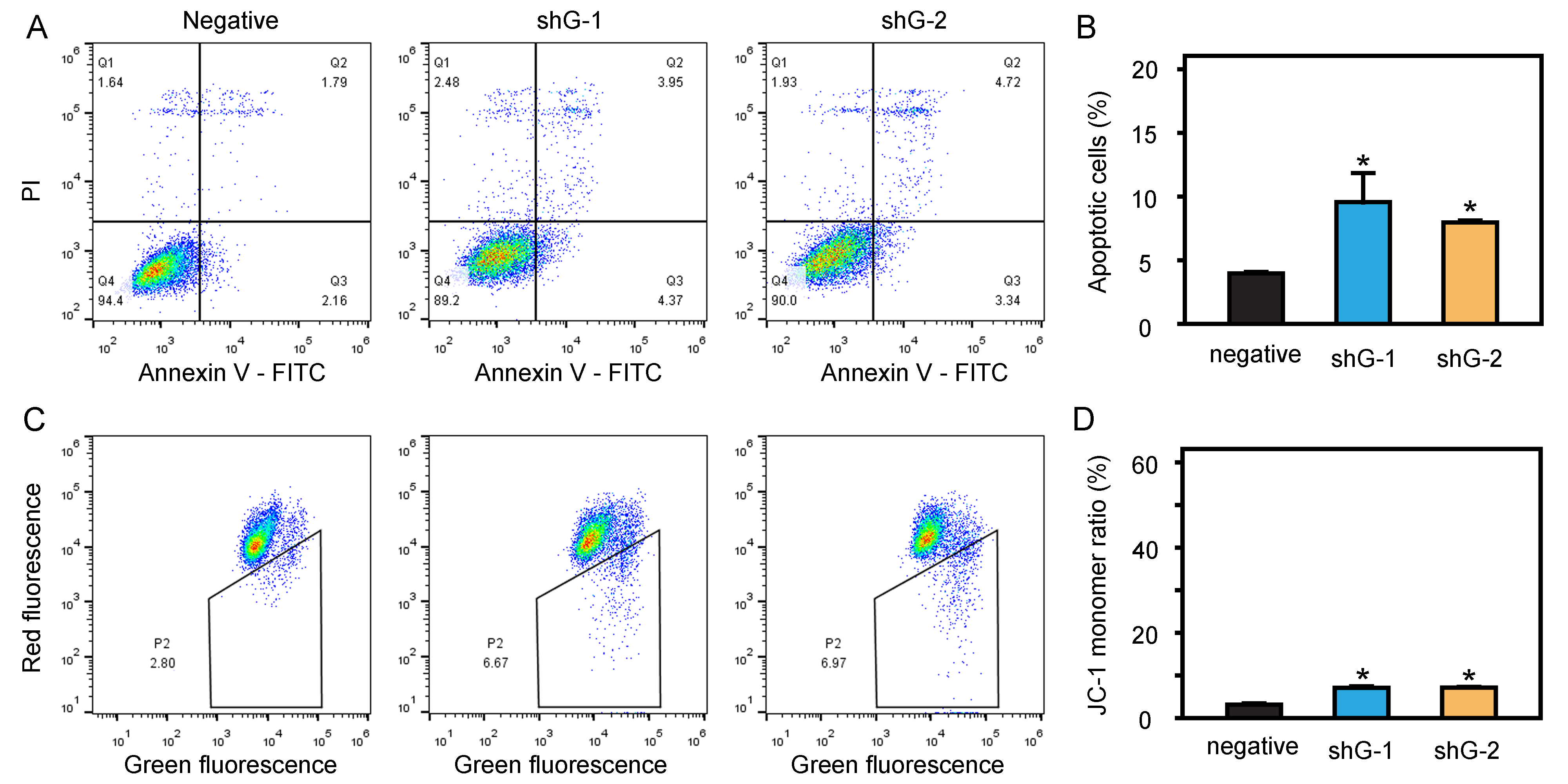

Supplement: S2 Fig — A) H460/DDP cells transfected with GLIPR1 shRNA or negative shRNA in the absence of DDP were stained with FITC-annexin V/PI, and then analyzed by flow cytometry. B) The statistical analysis revealed that shG-1 or shG-2 significantly increased the apoptosis of H460/DDP cells compared to the negative control. The data are representative of three similar experiments. * indicates a significant difference at p < 0.05 versus the negative control. C) Representative histograms showing flow cytometry analysis of JC-1 staining. D) The statistical analysis revealed that shG-1 or shG-2 significantly increased the JC-1 monomer ratio of H460/DDP cells compared to the negative control in the absence of DDP. The data are representative of three similar experiments. * indicates a significant difference at p < 0.05 versus the negative control. (TIF) [file pone.0182410.s002.tif]
